# EGFR-TKI-associated colitis in non-small cell lung cancer: a retrospective characterization of clinical, radiographic, endoscopic, and histopathologic features

**DOI:** 10.3389/fphar.2026.1935622

**Published:** 2026-10-12

**Authors:** Shujun Wang, Chen Tan, Pufang Shi, Ya Li, Hefeng Zhan, Jiajing Li

**Affiliations:** 1 Department of Gastroenterology, The First Affiliated Hospital of Zhengzhou University, Zhengzhou, China; 2 Department of Radiology, The First Affiliated Hospital of Zhengzhou University, Zhengzhou, China; 3 Department of Pathology, The First Affiliated Hospital of Zhengzhou University, Zhengzhou, China

**Keywords:** clinical imaging, colitis, diarrhea, drug targets, drug-associated colitis, EGFR-TKIs, lung cancer, NSCLC

## Abstract

**Objective:**

Epidermal growth factor receptor tyrosine kinase inhibitors (EGFR-TKIs) are standard targeted therapies for EGFR-mutant non-small cell lung cancer (NSCLC), but clinically documented EGFR-TKI-associated colitis remains incompletely characterized. We aimed to describe its clinical, radiographic, endoscopic, histopathologic, management, and exploratory survival features.

**Methods:**

We conducted a single-center retrospective case-control analysis nested within an institutional cohort of 3,285 patients with NSCLC treated with EGFR-TKIs between January 2018 and July 2025. The analysis included 43 patients with documented radiographic and/or endoscopic colitis and 223 selected asymptomatic controls. Clinical, imaging, colonoscopic, histopathologic, treatment, and survival data were reviewed. Ever-smoking was examined in an exploratory univariable logistic analysis. Drug-specific EGFR-TKI odds were not estimated because a common time-aligned exposure definition for cases and controls could not be reconstructed, and overall survival was examined exploratorily.

**Results:**

Clinically documented EGFR-TKI-associated colitis occurred in 43/3,285 treated patients (1.3%). Ever-smoking was more frequent among cases than selected controls (39.5% vs. 20.2%; univariable odds ratio = 2.586, 95% confidence interval: 1.293–5.173, P = 0.007). Drug-specific odds were not estimated because a common time-aligned exposure definition could not be reconstructed. Median symptom onset was 3 months. Diarrhea occurred in 40/43 patients (93.0%); three patients had grade 0 diarrhea. Among 39 patients undergoing colonoscopy, 14 had normal-appearing mucosa; 11 of these patients underwent biopsy and showed histologic inflammation. Of 35 classifiable biopsies, 10 (28.6%) showed a microscopic pattern, 14 (40.0%) an inflammatory/IBD-like pattern, and 11 (31.4%) an ischemic-like pattern. In exploratory survival analysis, no statistically significant association between documented colitis and overall survival was detected (log-rank P = 0.538; adjusted HR = 1.184, 95% CI: 0.782–1.793, P = 0.426).

**Conclusion:**

Clinically documented EGFR-TKI-associated colitis showed heterogeneous radiographic, endoscopic, and histopathologic patterns. Normal-appearing mucosa did not exclude histologic inflammation in biopsied patients. The 1.3% value represents documented cases in this institutional cohort rather than population incidence. The recorded smoking difference, severity-stratified findings, treatment outcomes, and survival estimates should be interpreted as exploratory; no drug-specific colitis association is inferred.

## Introduction

Lung cancer remains the most commonly diagnosed cancer and the leading cause of cancer-related mortality worldwide, with non-small cell lung cancer (NSCLC) accounting for approximately 85% of cases (Fu et al., 2022). According to 2022 global cancer statistics, lung cancer was responsible for an estimated 1.8 million deaths, constituting 18.7% of all cancer-related fatalities (Bray et al., 2024). Despite advances in early detection and multimodal therapies, the prognosis for advanced-stage disease remains poor, underscoring the urgent need for targeted therapeutic strategies.

The introduction of epidermal growth factor receptor tyrosine kinase inhibitors (EGFR-TKIs) —including first-generation (gefitinib, erlotinib, icotinib), second-generation (afatinib), and third-generation (osimertinib) agents—has fundamentally transformed the therapeutic landscape for patients with EGFR-mutant NSCLC, significantly improving progression-free survival (PFS) and overall survival (OS) (Zhou et al., 2025), along with a lower incidence of systemic toxicities, including myelosuppression (Hsu et al., 2018). Consequently, these agents have become the standard first-line treatment for EGFR-mutant NSCLC (Zhou et al., 2025; Hsu et al., 2018).

Despite their favorable systemic safety profile, gastrointestinal (GI) adverse events, particularly diarrhea, are common and dose-limiting toxicities of EGFR-TKIs, affecting approximately 50%–90% of treated patients (Zhao et al., 2021; Mok et al., 2021; Li and Gu, 2019). Inhibition of EGFR signaling in healthy intestinal epithelial cells impairs mucosal homeostasis, reduces chloride absorption, and induces secretory diarrhea (Uribe et al., 1996). Severe diarrhea may necessitate TKI dose reduction or treatment discontinuation, potentially compromising therapeutic efficacy and survival benefits (Li and Gu, 2019). In addition, persistent diarrhea increases healthcare costs, nutritional deficiencies, electrolyte imbalances, and secondary complications such as dehydration, further diminishing patients’ quality of life.

Although case reports have described EGFR-TKI-associated enterocolitis (Xiao et al., 2021; Shujun et al., 2022; Takenaka et al., 2017; Ando et al., 2015; Bai et al., 2013), systematic analyses characterizing the severity, endoscopic features, and histopathologic patterns of these GI toxicities are lacking. Integrated evidence linking diarrhea severity, endoscopic findings, histopathologic patterns, and management remains limited. This knowledge gap hinders the optimization of supportive care and risk stratification for affected patients. This retrospective study aims to address this gap by analyzing the clinical, endoscopic, and therapeutic profiles of EGFR-TKI-associated diarrhea and colitis, providing actionable insights to improve patient management.

## Methods

### Study design and patient selection

This single-center, retrospective nested case-control study was conducted at the First Affiliated Hospital of Zhengzhou University and approved by the Institutional Review Board (approval number: 2025-KY-1492). The requirement for written informed consent was waived by the IRB owing to the retrospective, anonymized nature of the medical record review; consent from legal guardians or next of kin was neither obtained nor required. The study complied with the Declaration of Helsinki and the STROBE reporting guidelines.

We screened 3,285 consecutive patients with cytologically or histopathologically confirmed NSCLC who received EGFR-TKI therapy between January 2018 and July 2025 (Figure 1). EGFR mutation status (e.g., exon 19 deletion, L858R) was evaluated via ARMS-PCR or next-generation sequencing.

**FIGURE 1:**
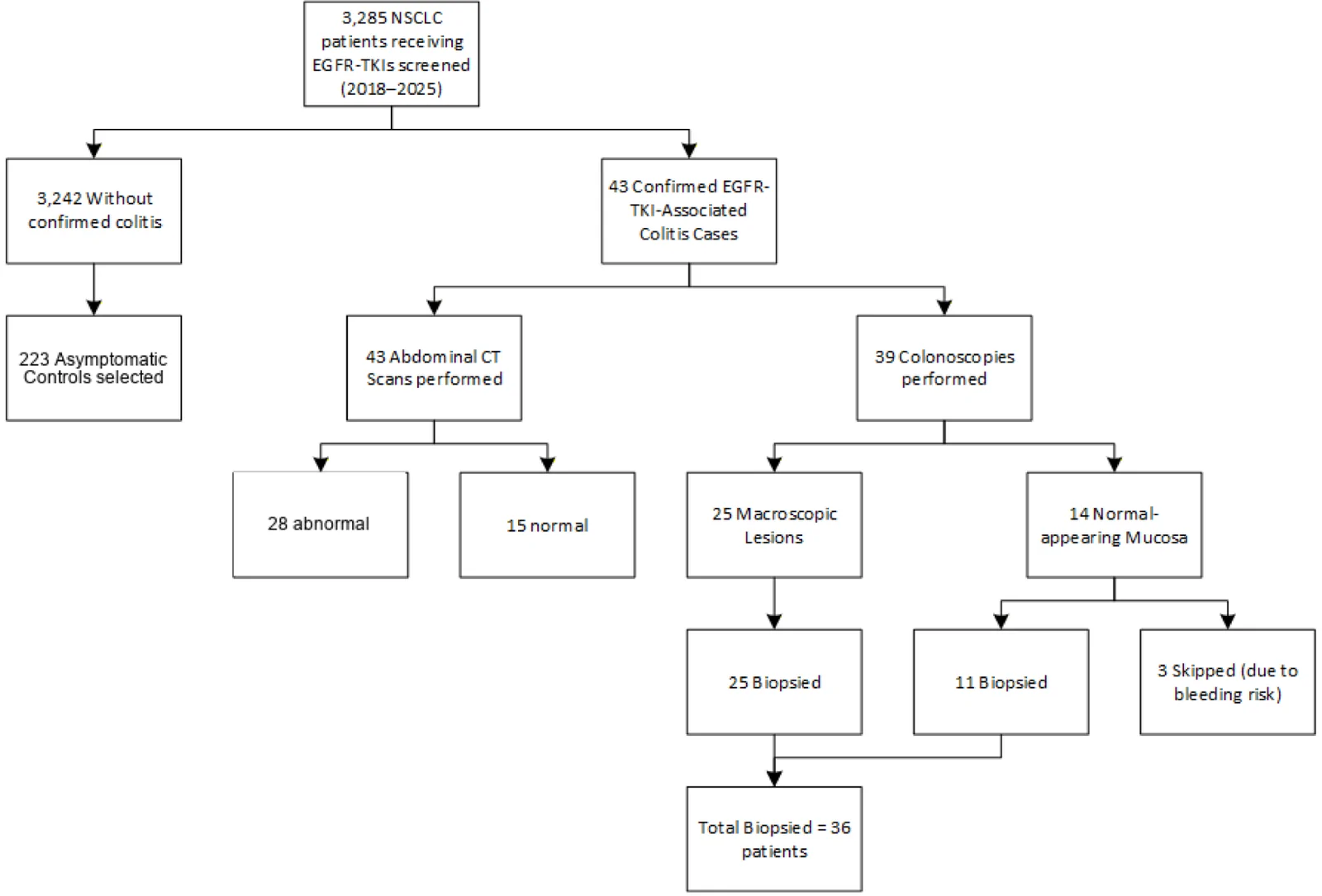
Patient selection flowchart. The 223 asymptomatic controls were selected from the treated cohort; a reproducible random-selection procedure could not be verified.

Patients were included as cases if they developed lower gastrointestinal symptoms (diarrhea, abdominal pain, or rectal bleeding) during EGFR-TKI therapy and had colitis documented by abdominal CT and/or endoscopy. Exclusion criteria were: (1) pre-existing inflammatory bowel disease or radiation enteritis; (2) active gastrointestinal infection identified, when testing was performed, by stool bacterial culture, Clostridioides difficile toxin PCR, or tissue CMV/EBV testing within 4 weeks of symptom onset; and (3) concurrent immune checkpoint inhibitor therapy or another treatment known to cause colitis. Infectious testing was ordered at the treating clinician’s discretion, and reliable patient counts for stool bacterial culture, *C. difficile* testing, and CMV/EBV testing could not be reconstructed from the available analytic extract. Previous immune checkpoint inhibitor exposure was also not consistently captured; only concurrent exposure was an exclusion criterion. The analytic control set comprised 223 eligible EGFR-TKI-treated patients from the same institutional period who remained free of gastrointestinal symptoms (CTCAE grade 0). Controls were sampled at approximately five per case; the retrospective sampling record did not retain enough documentation to verify or reproduce a random selection procedure. The final analysis included 43 cases and 223 selected controls.

### Data collection and treatment protocols

Data were retrospectively collected from institutional electronic medical records. Standard institutional starting regimens were gefitinib 250 mg once daily, erlotinib 150 mg once daily, icotinib 125 mg three times daily, afatinib 40 mg once daily, and osimertinib 80 mg once daily. Treatment was continued until progression, unacceptable toxicity, or withdrawal, with dose interruption or reduction according to the prescribing information and clinical judgment. For cases, the EGFR-TKI active at symptom onset was used for exposure classification. Because controls had no symptom-onset index date, they were classified by the last EGFR-TKI recorded during observation. Sequential therapy denoted successive, not concurrent, EGFR-TKI use. These non-time-aligned exposure categories were retained for description only and were not used to estimate drug-specific odds.

Demographics, tumor TNM stage (retrospectively according to TNM classification ninth edition (Asamura et al., 2023; Rami-Porta et al., 2024)), smoking status (never, former, current), concurrent medications, and laboratory parameters at colitis presentation (WBC, HGB, PLT, ESR, hsCRP) were extracted. Diarrhea severity was graded according to the National Cancer Institute’s Common Terminology Criteria for Adverse Events (CTCAE), version 5.0: (Freites-Martinez et al., 2021). Grade 1, increase of <4 stools per day over baseline; Grade 2, increase of 4–6 stools per day; Grade 3, increase of ≥7 stools per day, hospitalization indicated; Grade 4, life-threatening consequences.

### Radiographic, endoscopic and histopathologic evaluation

Contrast-enhanced abdominal/pelvic CT scans were evaluated for pericolonic fat stranding, colonic mural thickening (>3 mm), and luminal stenosis (defined as luminal narrowing with localized mural thickening). Endoscopic mucosal extent was classified as left-sided (distal to splenic flexure), right-sided (proximal to splenic flexure), extensive (both proximal and distal to the splenic flexure), or rectal. Mucosal features documented included erythema, erosions, ulcerations, or normal-appearing mucosa. Histopathological mucosal biopsies were reviewed by an experienced gastrointestinal pathologist. Colitis was classified according to standard diagnostic criteria (Hamdeh et al., 2021): (1) Microscopic colitis: defined as endoscopically normal or near-normal mucosa accompanied by histological intraepithelial lymphocytosis (>20 IELs per 100 surface epithelial cells) or a thickened subepithelial collagen band (>10 µm); (2) Inflammatory/IBD-like pattern: defined as endoscopic mucosal erythema, erosions, or ulcerations with histological crypt architectural distortion and active lamina propria inflammation; and (3) Ischemic-like pattern (Zou et al., 2009): defined as segmental, sharply demarcated mucosal edema, hemorrhage, superficial necrosis, or stromal hyalinization.

### Management and outcomes

Data on colitis management were collected, including EGFR-TKI modifications (dose reduction or discontinuation), use of supportive care, and specific treatments such as 5-aminosalicylic acid or systemic corticosteroids. Clinical response to treatment and colitis-related outcomes, including mortality, were documented. Survival status was ascertained via either telephone follow-up or clinical data review.

### Statistical analysis

Statistical analyses were performed using SPSS version 21.0 and independently verified against the analytic dataset. No formal *a priori* sample-size calculation was performed because this retrospective study included all eligible documented cases; no *post hoc* power calculation was reported. No values were missing for variables included in the reported tables and regression models. Continuous variables are reported as medians (interquartile ranges) and were compared using the Mann-Whitney U test. Categorical variables are reported as counts (percentages) and were compared using the chi-square or Fisher exact test, as appropriate. Because laboratory values in cases were measured at colitis presentation, they were treated as contemporaneous clinical correlates and were not entered as pretreatment predictors. Ever-smoking versus never-smoking was examined in an exploratory univariable logistic model, with the odds ratio (OR) and 95% confidence interval (CI) reported. Drug-specific EGFR-TKI logistic regression was not retained because cases and controls lacked a common time-aligned exposure definition. Overall survival was measured from EGFR-TKI initiation to death or administrative censoring on 31 July 2025. Kaplan-Meier curves, the log-rank test, and an exploratory Cox model including documented colitis, sex, stage, ECOG performance status, and NSAID use were used. Two-sided P < 0.05 was considered statistically significant; values below 0.001 are reported as P < 0.001.

## Results

### Documented frequency and baseline characteristics

Among 3,285 NSCLC patients treated with EGFR-TKIs, 43 developed confirmed colitis, representing a documented frequency of 1.3%. The administrative extract contained one EGFR-TKI category per cohort patient, but did not retain enough provenance to determine whether that category represented first treatment, final treatment, or a time-matched index exposure; the ambiguous full-cohort drug counts were therefore removed from the revised presentation.

Baseline demographic and clinical characteristics were similar between cases (n = 43) and selected controls (n = 223) for age, sex, tumor stage, ECOG performance status, and recorded comorbidities (Table 1). Ever-smoking was more frequent among cases (39.5% vs. 20.2%, P = 0.006). Laboratory values in cases were measured during active colitis and showed higher WBC, ESR, and hsCRP and lower hemoglobin than values recorded for controls. These contemporaneous differences may reflect inflammation, bleeding, or dehydration and were not interpreted as pretreatment risk factors.

**TABLE 1:** Comparison of demographic and clinical characteristics and laboratory values at assessment between patients with and without EGFR-TKI-associated colitis.

Parameter	Total (N = 266)	Colitis group (N = 43)	Control group (N = 223)	P-value
Male sex, n (%)	160 (60.2)	24 (55.8)	136 (61)	0.526
Median age, years (IQR)	61 (55–68.3)	60 (55–68)	61 (55–69)	0.659
TNM stage (IV/III), n	175/91	29/14	146/77	0.803
ECOG performance status (0–1/≥2)	240/26	37/6	203/20	0.467
Smoking status, n (%)	​	​	​	**0.022**
Never smoker	204 (76.7)	26 (60.5)	178 (79.8)	​
Former smoker	38 (14.3)	10 (23.3)	28 (12.6)	​
Current smoker	24 (9.0)	7 (16.3)	17 (7.6)	​
Smoking history, n (%)	62 (23.3)	17 (39.5)	45 (20.2)	**0.006**
NSAID use (past 3 months), n (%)	19 (7.1)	4 (9.3)	15 (6.7)	0.782
Prior chemotherapy, n (%)	66 (24.8)	10 (23.3)	56 (25.1)	0.796
Hypertension, n (%)	84 (31.6)	11 (25.6)	73 (32.7)	0.355
Coronary heart disease, n (%)	34 (12.8)	6 (14)	28 (12.6)	0.802
Laboratory values at assessment				
WBC (10^9/L), median (IQR)	5.16 (4.20–6.70)	7.50 (5.20–9.30)	4.96 (4.18–6.13)	**<0.001**
Hemoglobin (g/L), median (IQR)	123.0 (110–135)	95.0 (83–105)	126.4 (116–136)	**<0.001**
Platelets (10^9/L), median (IQR)	186.5 (141.5–241.3)	199 (98–289)	185 (146–233)	0.900
ESR (mm/h), median (IQR)	15.2 (9.8–19.7)	28 (15–42)	14.1 (9.4–18.7)	**<0.001**
hsCRP (mg/L), median (IQR)	4.37 (1.26–8.92)	35.6 (9.0–55.2)	3.09 (0.97–6.71)	**<0.001**

Abbreviations: ECOG, eastern cooperative oncology group; ESR, erythrocyte sedimentation rate; hsCRP, high-sensitivity C-reactive protein; IQR, interquartile range; NSAID, non-steroidal anti-inflammatory drug; WBC, white blood cell count. Bold values indicate statistical significance (P < 0.05).

### EGFR-TKI regimens and exposure assignment

The recorded EGFR-TKI categories are shown descriptively in Table 2. Cases were classified by the EGFR-TKI active at symptom onset, whereas controls, who had no symptom-onset index date, were classified by the last EGFR-TKI recorded during observation. Four cases and 37 controls had sequential therapy ending on osimertinib. Because the exposure definitions and time points were not directly comparable, no between-group exposure test or drug-specific logistic regression was retained. The ambiguous full-cohort category totals were also removed because their first, final, or index-treatment meaning could not be verified.

**TABLE 2:** Recorded EGFR-TKI categories in cases and selected controls (descriptive, non-time-aligned classifications).

EGFR-TKI regimen	Cases: active at symptom onset (n = 43)	Controls: last recorded regimen (n = 223)	Assignment note
Afatinib	20 (46.5%)	22 (9.9%)	-
Erlotinib	9 (20.9%)	23 (10.3%)	-
Icotinib	4 (9.3%)	37 (16.6%)	-
Osimertinib	8 (18.6%)*	108 (48.4%)**	See footnotes
Gefitinib	2 (4.7%)	33 (14.8%)	-

* Includes 4 sequentially treated cases; classification used the EGFR-TKI active at symptom onset.

** Includes 37 sequentially treated controls; because no symptom-based index date existed, classification used the last recorded EGFR-TKI.

The drug-specific logistic model and afatinib odds ratio were removed because cases and controls did not have a common time-aligned EGFR-TKI exposure definition. Ever-smoking was examined separately in an exploratory univariable analysis and was more frequent among cases (OR = 2.586, 95% CI: 1.293–5.173, P = 0.007; Table 3). Diagnosis-time laboratory values remained excluded because they were not temporally suitable as pretreatment predictors.

**TABLE 3:** Exploratory univariable analysis of ever-smoking and documented EGFR-TKI-associated colitis.

Variable	OR (95% CI)	P-value	Analysis	Interpretation
Ever-smoking vs. never-smoking	2.586 (1.293–5.173)	0.007	Univariable logistic	Exploratory

### Clinical and radiographic features

The median onset time of colitis was 3 months (IQR 2–6 months) after initiation of the EGFR-TKI active at symptom onset. Diarrhea occurred in 40 patients (93.0%), abdominal pain in 15 (34.9%), and rectal bleeding in 13 (30.2%). Median symptom duration was 12 days (IQR 9–30 days). Across all 43 cases, 3 patients (7.0%) had grade 0 diarrhea, 5 (11.6%) grade 1, 23 (53.5%) grade 2, 8 (18.6%) grade 3, and 4 (9.3%) grade 4. No grade 5 colitis-related death was recorded (Table 4).

**TABLE 4:** Clinical and CT characteristics of colitis patients.

Characteristic	Value [n (%) or median (IQR)]
Median onset time, months (IQR)	3 (2–6)
Clinical presentation	
Diarrhea	40 (93.0)
Abdominal pain	15 (34.9)
Rectal bleeding	13 (30.2)
CTCAE Diarrhea grade (n=43)	
Grade 0 (pain/bleeding without diarrhea)	3 (7.0)
Grade 1	5 (11.6)
Grade 2	23 (53.5)
Grade 3	8 (18.6)
Grade 4	4 (9.3)
Median symptom duration, days (IQR)	12 (9–30)
Abdominal CT features	
Abnormal CT findings	28 (65.1)
Colonic wall thickening	24 (55.8)
Pericolonic fat stranding	20 (46.5)
Luminal stenosis	9 (20.9)

Abdominal CT scans revealed abnormal findings in 28 patients (65.1%). The most frequent signs were colonic wall thickening (55.8%), pericolonic fat stranding (46.5%), and luminal stenosis (20.9%) (Figure 2).

**FIGURE 2:**
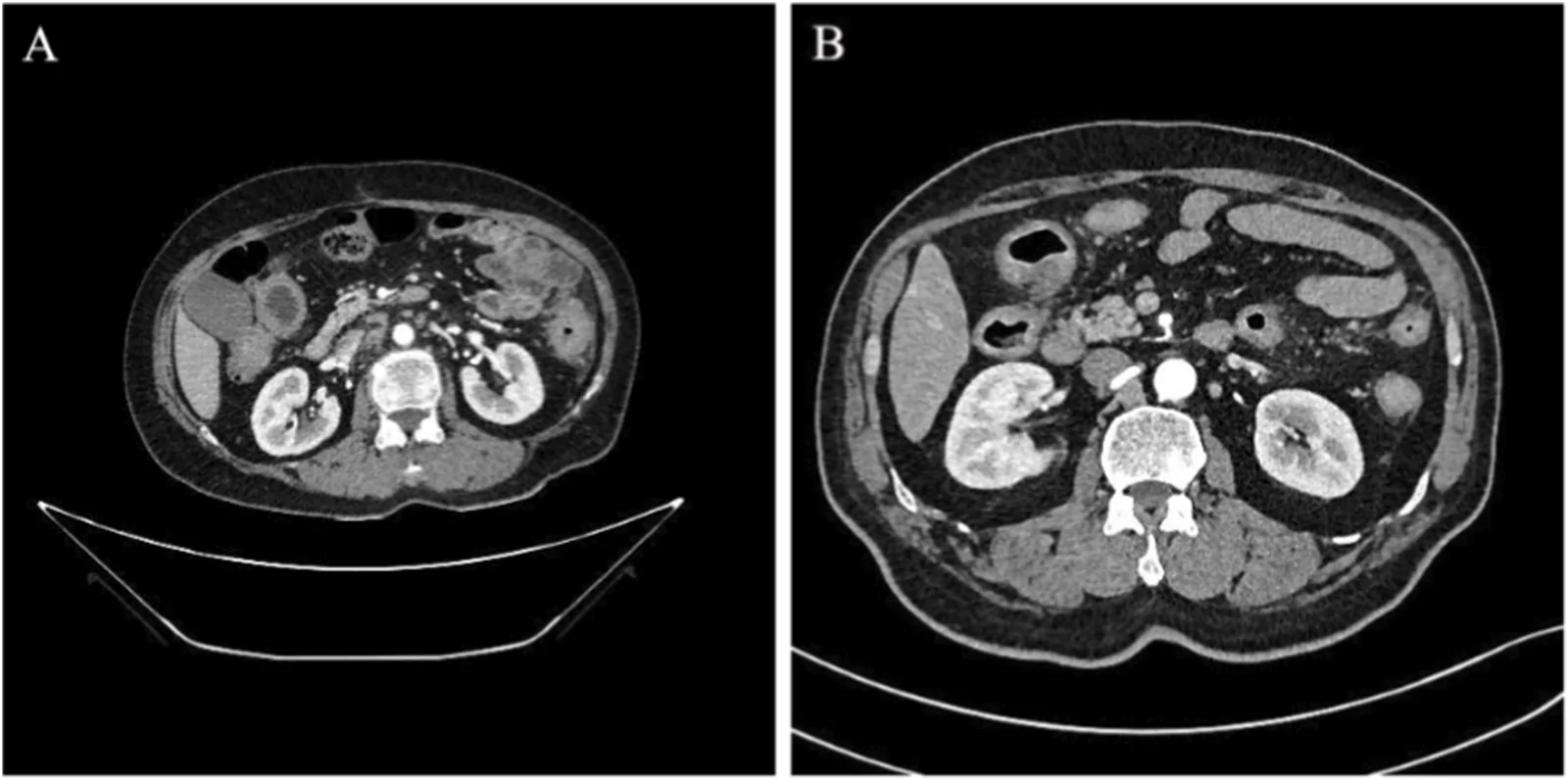
CT features of colitis. **(A)** Colonic wall thickening and luminal narrowing of the ascending and descending colon. **(B)** Colonic wall thickening and pericolonic fat stranding.

### Endoscopic and histopathologic spectrum

Colonoscopy was performed in 39/43 patients. Four did not undergo colonoscopy because of hemodynamic instability (n = 2), severe heart failure (n = 1), or refusal (n = 1). Among the 39 examined patients, 25 (64.1%) had macroscopic mucosal abnormalities: erythema in 25 (64.1%), erosions in 22 (56.4%), and ulceration in 15 (38.5%; 13 superficial and 2 deep) (Figure 3). Involvement was left-sided in 12 (30.8%), extensive in 8 (20.5%), right-sided in 3 (7.7%), and isolated to the rectum in 2 (5.1%). Fourteen patients (35.9%) had normal-appearing mucosa.

**FIGURE 3:**
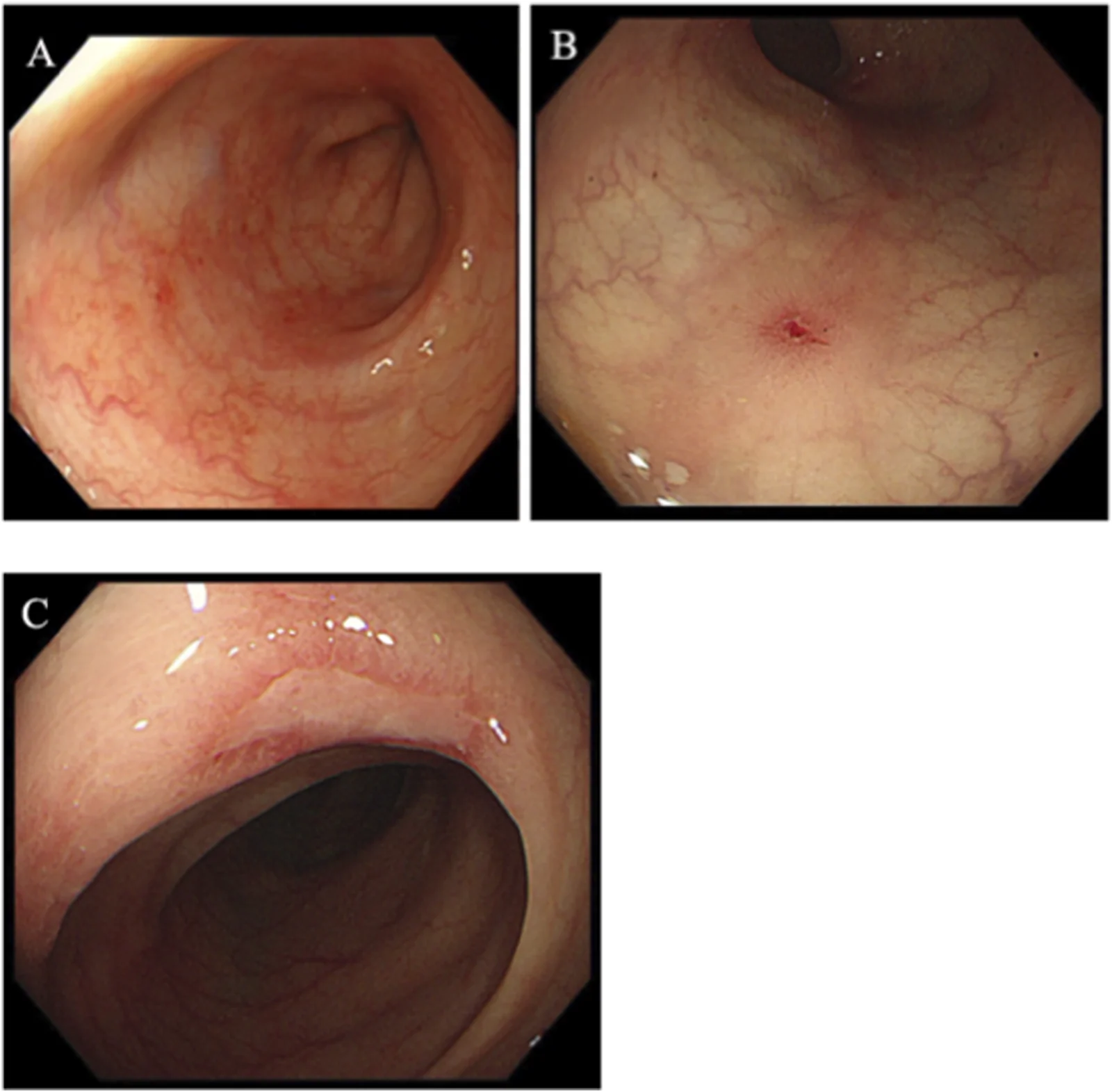
Endoscopic features of colitis. **(A)** Patchy erythema. **(B)** Erosion. **(C)** Superficial ulceration.

Mucosal biopsies were available for 36 patients (Table 5). All 25 patients with macroscopic lesions underwent biopsy. Of 14 patients with normal-appearing mucosa, 11 underwent biopsy and showed histologic inflammation; 3 were not biopsied because of bleeding risk. Among the 36 biopsies, intraepithelial lymphocytosis was reported in 31 (86.1%), crypt architectural distortion in 24 (66.7%), granulocytic infiltration in 21 (58.3%), and eosinophilic infiltration in 17 (47.2%) (Figure 4). Biopsy sites and microscopic-colitis subtypes were not consistently retrievable, and formal blinded duplicate pathology adjudication was not performed.

**TABLE 5:** Endoscopic, radiographic, histopathologic, and treatment findings stratified by CTCAE diarrhea severity.

Feature	Overall	Grade 0–2	Grade 3–4	P-value
Endoscopic mucosa (colonoscopy n = 39)	​	n = 28	n = 11	0.060
Normal-appearing mucosa	14/39 (35.9)	13/28 (46.4)	1/11 (9.1)	​
Macroscopic abnormality	25/39 (64.1)	15/28 (53.6)	10/11 (90.9)	​
CT wall thickening (CT n = 43)	24/43 (55.8)	13/31 (41.9)	11/12 (91.7)	0.005
Classifiable biopsy pattern (n = 35)	​	n = 24	n = 11	0.212
Microscopic pattern	10/35 (28.6)	9/24 (37.5)	1/11 (9.1)	​
Inflammatory/IBD-like pattern	14/35 (40.0)	8/24 (33.3)	6/11 (54.5)	​
Ischemic-like pattern	11/35 (31.4)	7/24 (29.2)	4/11 (36.4)	​
Nonspecific/unclassified biopsy (n = 36)	1/36 (2.8)	1/25 (4.0)	0/11 (0.0)	-
Systemic corticosteroid use (n = 43)	12/43 (27.9)	1/31 (3.2)	11/12 (91.7)	<0.001

**FIGURE 4:**
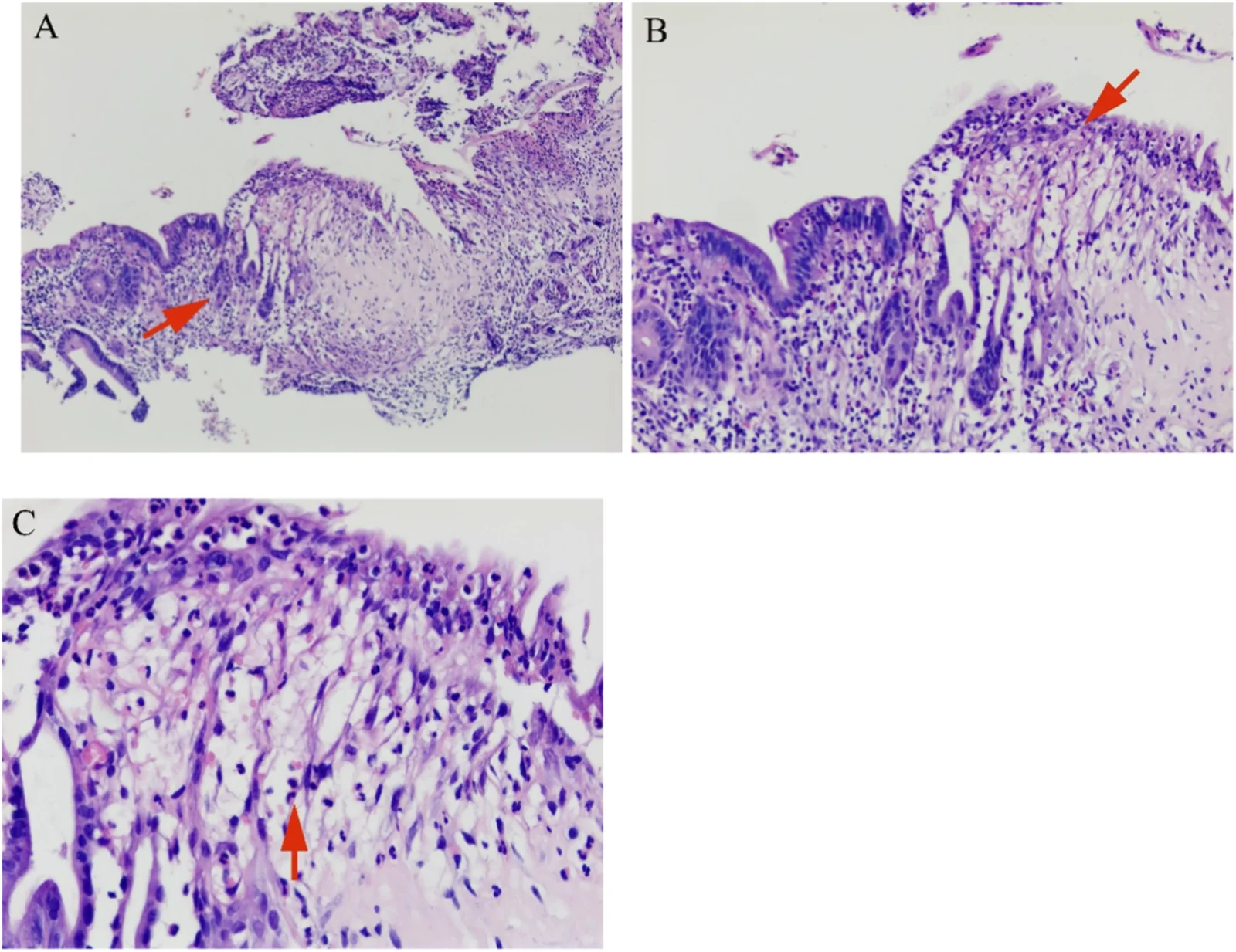
Histopathologic features. **(A)** Chronic inflammatory change with crypt architectural distortion (red arrow; H&E, original magnification 100×). **(B)** Intraepithelial lymphocytosis and granulocytic infiltration (red arrow; H&E, 200x). **(C)** Eosinophilic infiltration (red arrow; H&E, 400x). Calibrated scale bars were not added because calibration metadata were unavailable for the archived images.

Of 36 biopsied patients, 35 had a classifiable histologic pattern: microscopic in 10/35 (28.6%), inflammatory/IBD-like in 14/35 (40.0%), and ischemic-like in 11/35 (31.4%). One additional biopsy (1/36, 2.8%) showed nonspecific mild inflammation and remained unclassified (Table 5). These labels describe compatible patterns and do not by themselves establish a specific etiology.

### Management and survival outcomes

The 31 patients with grade 0–2 disease received supportive treatment as clinically indicated; the 28 patients with grade 1–2 diarrhea received oral loperamide or racecadotril, probiotics, and fluid replacement. All 12 patients with grade 3–4 disease underwent EGFR-TKI interruption and intravenous fluid replacement. Systemic corticosteroids were given to 12 patients overall: 1/31 with grade 0–2 disease and 11/12 with grade 3–4 disease (intravenous methylprednisolone 1 mg/kg/day or oral prednisone 0.75 mg/kg/day for 2 weeks, followed by a 4-week taper). Eight patients with fever or neutropenia received empiric antibiotics for 7–10 days. After improvement to grade 1 or lower, 12 patients were rechallenged with an EGFR-TKI (8 at a reduced dose and 4 after switching to osimertinib); no recurrence was recorded during a median post-rechallenge follow-up of 8 months. The available extract did not retain the specific restarted agents and reduced doses for the eight dose-reduced patients or whether systemic corticosteroids were still being administered at the time of restart. These uncontrolled retrospective observations describe management and recurrence but do not establish treatment effectiveness or safety equivalence.

In exploratory survival analysis, median OS was 14.0 months (95% CI: 8.86–19.14) among cases and 24.5 months (95% CI: 19.33–29.67) among selected controls (log-rank P = 0.538; Figure 5). There were 28 deaths among 43 cases and 135 among 223 controls; reverse Kaplan-Meier median follow-up was 36.0 months. In the reproducible Cox model including documented colitis, sex, stage, ECOG performance status, and NSAID use, colitis was not significantly associated with OS (adjusted HR = 1.184, 95% CI: 0.782–1.793, P = 0.426; Table 6). Male sex, stage IV disease, and NSAID use were associated with poorer OS. Because colitis was not modeled as a time-dependent exposure and controls were selected, these analyses are exploratory and do not demonstrate equivalence or absence of an effect.

**FIGURE 5:**
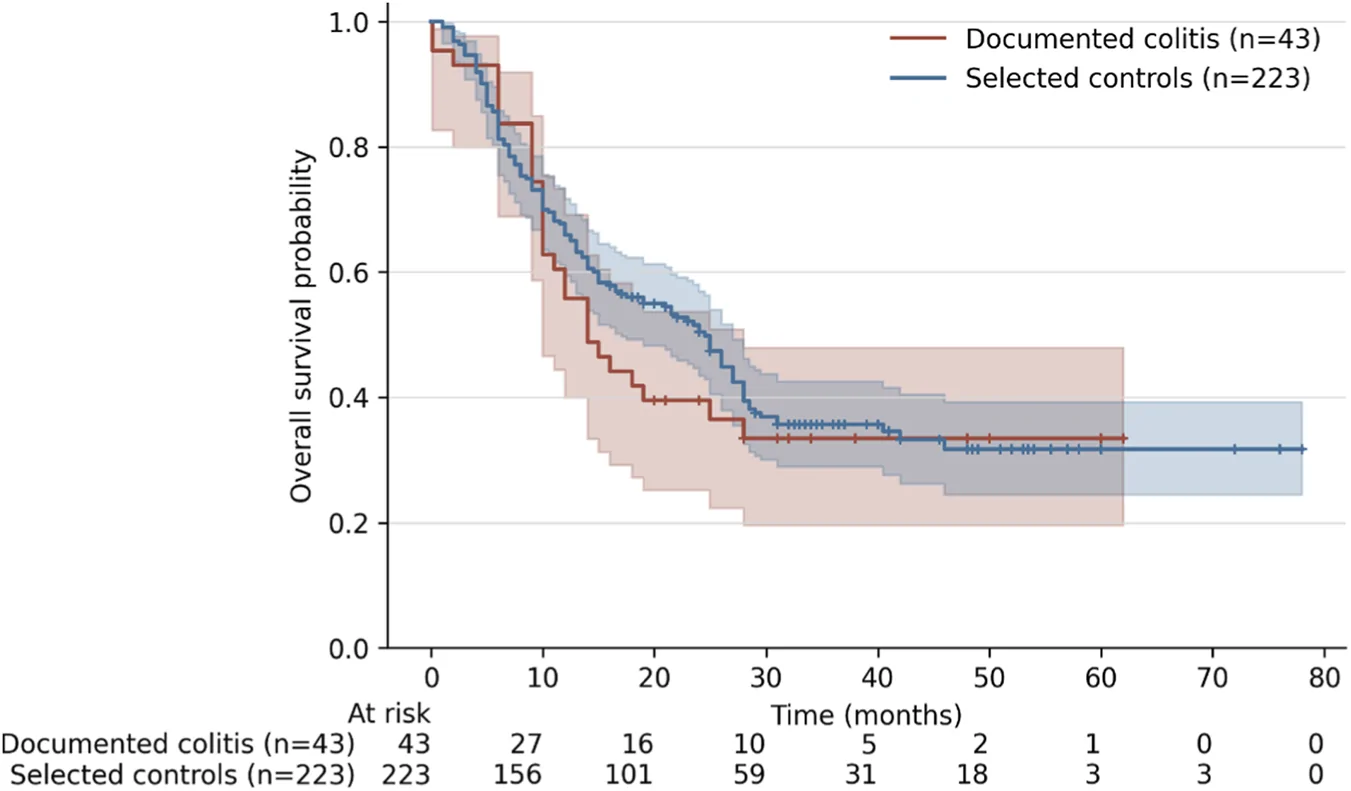
Exploratory Kaplan-Meier curves for overall survival in documented colitis cases and selected asymptomatic controls. Shaded areas indicate 95% confidence intervals; plus signs indicate censoring. Numbers at risk are shown below the plot.

**TABLE 6:** Exploratory Cox regression analysis of overall survival.

Variable	Univariable HR (95% CI)	P-value	Adjusted HR (95% CI)	P-value
Documented colitis (yes vs. no)	1.144 (0.761–1.719)	0.518	1.184 (0.782–1.793)	0.426
Male sex	1.940 (1.385–2.716)	<0.001	1.897 (1.349–2.667)	<0.001
Stage IV (vs. stage III)	1.559 (1.113–2.185)	0.010	1.510 (1.071–2.127)	0.019
ECOG PS >=2 (vs. 0–1)	1.083 (0.636–1.842)	0.770	1.134 (0.658–1.954)	0.652
NSAID use	2.808 (1.689–4.669)	<0.001	2.323 (1.386–3.895)	0.001

## Discussion

EGFR-TKIs represent a cornerstone in the management of advanced NSCLC harboring activating EGFR mutations, significantly improving patient outcomes (Zhou et al., 2025). While diarrhea is a well-recognized and frequent adverse event associated with these agents (Li and Gu, 2019; Z et al., 2022; Yin et al., 2021; Takeda and Nakagawa, 2017) and often leads to dose interruption, detailed descriptions of intestinal symptoms and radiographic changes have been limited to previous case reports (Shujun et al., 2022; Bai et al., 2013; Oku et al., 2021; Kwok et al., 2018; Yamaguchi et al., 2018). Integrated real-world descriptions of the clinical, endoscopic, and histopathologic spectrum of EGFR-TKI-associated colitis remain limited. This study provides a detailed real-world description of colitis associated with commonly used EGFR-TKIs (afatinib, erlotinib, icotinib, osimertinib, gefitinib) in NSCLC patients. Our findings reveal that EGFR-TKI-associated colitis presents with a diverse range of clinical severities and pathological patterns, including microscopic, inflammatory (IBD-like), and ischemic-like patterns.

### Documented frequency and onset

The observed proportion of clinically documented EGFR-TKI-associated colitis in our institutional cohort was 1.3%, substantially lower than the reported rates of all-grade diarrhea in pivotal clinical trials and meta-analyses. Although the risk of all-grade diarrhea varies significantly across studies, ranging from 18% to 95%, up to 25% of patients experience grade ≥3 diarrhea (Li and Gu, 2019; Hirsh et al., 2014). This discrepancy likely reflects several factors: our study specifically focused on patients with confirmed colitis (often requiring endoscopic/histologic confirmation), whereas trials report patient-reported diarrhea symptoms; mild diarrhea cases may be managed in outpatient settings without extensive investigation and are therefore likely underrepresented in hospital-based retrospective studies. Additionally, selection bias toward more severe cases requiring hospitalization or specialized consultation may exist. Cross-study comparisons should be interpreted cautiously because populations and outcome definitions differ. The documented 1.3% proportion is numerically higher than that reported for taxane-induced colitis (0.2% in a large cohort) (Chen et al., 2020) and numerically lower than reported colitis rates associated with immune checkpoint inhibitors (ICIs) (up to 40% diarrhea and 2%–5% severe enterocolitis) (Dougan et al., 2021). It reflects the proportion of clinically recognized and documented cases in this cohort and should not be interpreted as the true population incidence.

Afatinib accounted for 20/43 cases and 22/223 controls in the recorded categories; however, cases were classified at symptom onset and controls by the last recorded EGFR-TKI, so these proportions are descriptive, not directly comparable, and are not drug-specific risks. This distribution is broadly consistent with prior diarrhea-toxicity reports, although direct comparison is limited (Hirsh et al., 2014; Hashimoto et al., 2023). Afatinib exhibited greater toxicity in cultured intestinal stem cells compared with other EGFR-TKIs (Hashimoto et al., 2023). In this study, EGFR-TKI-associated colitis did not occur immediately after treatment initiation but rather developed gradually, with a median onset of 3 months—longer than the reported onset of diarrhea (approximately 2 weeks after treatment initiation) (Asnacios et al., 2009). However, this timing aligns with previous case reports of intestinal symptoms, which ranged from 10 days to 12 months (Takenaka et al., 2017; Oku et al., 2021; Kwok et al., 2018; Uruga et al., 2018). This delay may reflect the time required for colonic or radiographic changes to become apparent.

### Clinical presentation and severity

Diarrhea was nearly universal (93%) among patients with confirmed colitis, consistent with its prominence as an EGFR-TKI side effect. However, the severity profile in our colitis cohort appeared skewed toward higher grades compared with overall diarrhea reported in trials, with the majority experiencing grade 2 (53.5%) or higher (grade 3–4: 27.9%) diarrhea. This may reflect the tertiary referral setting of our institution. Abdominal pain (35%) and rectal bleeding (30%) were also frequently observed in EGFR-TKI-associated colitis, features commonly seen in other DIC entities, including taxane- and ICI-related colitis (Chen et al., 2020; Lenti et al., 2024). Lower hemoglobin and higher ESR and hsCRP were observed at colitis diagnosis. Because these measurements were contemporaneous with active disease, bleeding, inflammation, and dehydration may explain the differences; they should not be interpreted as pretreatment predictors, but may prompt further clinical evaluation. On CT, colonic wall thickening was the most common finding (55.8%). Abnormal CT scans reflected colonic mucosal injury and subsequent inflammation. CT-reported luminal narrowing was observed in some cases, but its temporal relationship to the acute episode was uncertain. Intestinal perforation and toxic megacolon have been reported in case reports (Kwok et al., 2018; Yamaguchi et al., 2018), but were not observed in our study.

### Endoscopic and histopathological spectrum

A key finding of our study is the heterogeneity of endoscopic and histopathologic presentations of EGFR-TKI-associated colitis. Endoscopically, while erythema and erosions were common, a substantial proportion (38.5%) exhibited ulcerations; this proportion was numerically higher than that reported for ICIs (32%) (Geukes Foppen et al., 2018) and taxanes (27%) (Chen et al., 2020). Notably, 35.9% of symptomatic patients undergoing colonoscopy had normal-appearing mucosa, mimicking findings in taxane-induced colitis (31% normal) (Chen et al., 2020) and highlighting the necessity of biopsies even in the absence of visible lesions. This contrasts with typical ICI colitis, where macroscopic inflammation is more consistently observed (68%) (Geukes Foppen et al., 2018). The predominantly left-sided or extensive distribution is a pattern seen in various forms of colitis, including ulcerative colitis and ICI-induced colitis (Wright et al., 2019). Pathologic features of DIC are nonspecific (Herlihy and Feakins, 2022). Intraepithelial lymphocytosis (86.1%) and crypt architectural distortion (66.7%) were common but not sufficiently specific to distinguish among different DICs. Most histological findings consisted of inflammatory cell infiltration.

The exact mechanisms underlying EGFR-TKI-associated colitis remain incompletely understood. EGFR is expressed in both cancer cells and healthy gastrointestinal epithelial cells and plays critical roles in maintaining mucosal integrity, reducing bacterial translocation, and preserving gut barrier function after injury (Shujun et al., 2022; Van Sebille et al., 2017). Inhibition of EGFR signaling by TKIs may directly impair these functions. Several non-mutually exclusive mechanisms may contribute: loss of EGFR-mediated restraint of epithelial chloride secretion can promote secretory diarrhea; inhibition of epithelial proliferation and restitution can impair mucosal repair and tight-junction integrity; increased permeability may facilitate bacterial translocation and amplify innate inflammatory signaling; and treatment-related shifts in the intestinal microbiota may further modify barrier and immune responses (Ariyadamrongkwan and Muanprasat, 2025). First-generation EGFR-TKIs, such as gefitinib and erlotinib, reversibly inhibit EGFR kinases by blocking the ATP-binding site of EGFR kinases (Tao and Chityala, 2021). Second-generation EGFR-TKIs, such as afatinib, irreversibly inhibit both wild-type and mutant EGFR, and can produce more frequent or severe diarrhea than more selective agents (Hashimoto et al., 2023). In our sampled dataset, afatinib accounted for 20/43 documented colitis cases. Because a common time-aligned exposure definition could not be reconstructed, the afatinib odds estimate was removed and no drug-specific association is inferred from these recorded categories. Third-generation EGFR-TKIs, such as osimertinib, selectively target T790M mutants to overcome T790M-driven resistance, and accounted for 4/43 documented colitis cases in our sampled dataset; full-cohort drug-specific frequency could not be estimated, although prior studies report lower diarrhea rates (Li and Gu, 2019; Tao and Chityala, 2021).

### Diagnosis and management

Our study highlights the importance of a systematic approach to persistent or severe diarrhea in patients receiving EGFR-TKIs. While CT scans may reveal nonspecific signs such as wall thickening, colonoscopy is crucial for confirming colitis, assessing severity, and ruling out alternative diagnoses such as infection (especially *C. difficile*) or IBD exacerbation. The high rate of endoscopically normal mucosa in symptomatic patients underscores the need for routine biopsies. Management of EGFR-TKI-related diarrhea has been described in previous studies (Tao and Chityala, 2021; Cardenas-Fernandez et al., 2023). For grade 1–2 diarrhea, published guidance recommends hydration and loperamide 4 mg initially followed by 2 mg after each loose stool, up to the applicable daily maximum, whereas grade 3–4 diarrhea warrants temporary EGFR-TKI interruption, investigation for organic causes, and escalation of supportive care; corticosteroid use for confirmed inflammatory colitis should be individualized because effectiveness was not established by these uncontrolled observations (Tao and Chityala, 2021). The management of EGFR-TKI-associated diarrhea is, to some extent, similar to that of ICI colitis, suggesting potential overlap in downstream inflammatory pathways in severe cases.

### Prognostic factors for overall survival

No statistically significant association between documented colitis and overall survival was detected (median OS: 14.0 vs. 24.5 months; log-rank P = 0.538). There were 28 deaths among cases and 135 among controls, and reverse Kaplan-Meier median follow-up was 36.0 months. The numerically shorter median OS among cases and the limitations of selected controls and time-fixed exposure require cautious interpretation.

In the reproducible exploratory Cox model including documented colitis, sex, stage, ECOG performance status, and NSAID use, documented colitis was not significantly associated with OS (adjusted HR = 1.184, 95% CI: 0.782–1.793, P = 0.426). This result means only that no statistically significant association was detected; it does not demonstrate equivalence or absence of an effect.

Several covariates were associated with poorer prognosis in the exploratory model. Male sex was associated with worse OS (adjusted HR = 1.897, 95% CI: 1.349–2.667, P < 0.001), consistent with well-documented sex differences in NSCLC outcomes. Female patients with EGFR-mutant NSCLC may have longer survival in relation to higher EGFR mutation rates, more favorable responses to EGFR-TKIs, and potential hormonal influences (Yu et al., 2026; Yu et al., 2022). Advanced cancer stage (stage IV vs. III) was associated with poorer OS (adjusted HR = 1.510, 95% CI: 1.071–2.127, P = 0.019), reflecting the inherent aggressiveness of metastatic disease. Notably, NSAID use within 3 months was associated with poorer OS (adjusted HR = 2.323, 95% CI: 1.386–3.895, P = 0.001). While NSAIDs have been investigated for their controversial efficacy in patients with advanced NSCLC (Li et al., 2026; Roszik et al., 2022; Deng et al., 2025; Brasky et al., 2012), their use in advanced NSCLC patients may reflect underlying comorbidities or disease-related pain, rather than a direct adverse effect on survival. Given the retrospective nature of this analysis, this association should be interpreted cautiously and warrants further investigation in prospective studies.

### Strengths and limitations

The principal strength of this study is its detailed radiographic, endoscopic, and histopathologic description of a clinically important, under-recognized adverse event. Limitations include retrospective ascertainment; probable under-detection of mild cases; use of selected asymptomatic controls rather than the full treated cohort or symptomatic patients without objective colitis; inability to verify or reproduce the control-selection procedure; absence of a common time-aligned EGFR-TKI exposure definition for cases and controls; inability to verify whether the administrative full-cohort drug category represented first, final, or index treatment; incomplete and clinician-directed infectious testing with unavailable patient-level counts for stool bacterial culture, *C. difficile*, and CMV/EBV testing; unavailable reliable data on previous immune checkpoint inhibitor exposure; unavailable agent-specific reduced-dose restart regimens and corticosteroid status at rechallenge; absence of pack-years and some other patient-level exposure details; aggregate rather than case-level source availability for portions of the severity analysis; lack of consistently recorded biopsy sites, microscopic-colitis subtypes, duplicate pathology adjudication, and image-calibration metadata; contemporaneous rather than pretreatment laboratory measurements; limited events for regression; potential residual confounding; and possible immortal-time bias because colitis was not modeled as a time-dependent exposure. Baseline colonoscopy, biopsy, and imaging were not systematically available. Accordingly, the 1.3% value is a documented proportion, subtype labels are descriptive patterns, treatment outcomes are uncontrolled observations, and all case-control and survival estimates should be interpreted as exploratory.

## Conclusion and future directions

Clinically documented EGFR-TKI-associated colitis in this retrospective series showed heterogeneous radiographic, endoscopic, and histopathologic patterns, including microscopic, inflammatory/IBD-like, and ischemic-like appearances. The observed 1.3% represents recognized and documented cases in one institutional cohort and not the true incidence. Normal-appearing mucosa did not exclude histologic inflammation in the 11 patients who underwent biopsy. Clinicians should evaluate persistent or severe gastrointestinal symptoms for infectious, ischemic, medication-related, and other causes and consider biopsy even when mucosa appears normal. Because a common time-aligned EGFR-TKI exposure definition could not be reconstructed, no drug-specific colitis association is inferred. The observed smoking difference and survival analyses remain exploratory; prospective full-cohort studies with time-dependent exposure modeling and standardized diagnostic and management protocols are needed.

## Data Availability

The original contributions presented in the study are included in the article/Supplementary Material, further inquiries can be directed to the corresponding author.
