# Supplementary material for: EGFR-TKI-associated colitis in non-small cell lung cancer: a retrospective characterization of clinical, radiographic, endoscopic, and histopathologic features
Source: Front Pharmacol. 2026 Oct 12;17:1935622. doi: 10.3389/fphar.2026.1935622 (PMC13696202; doi:10.3389/fphar.2026.1935622)
Supplement: Supplementary file 1 [file Supplementaryfile1.docx]

**Supplementary Table. Univariate and multivariable Cox regression analysis of factors associated with overall survival.**

| Variables | Univariate analysis | | Multivariable analysis | |
| --- | --- | --- | --- | --- |
|  | HR (95% CI) | P | HR (95% CI) | P |
| Colitis (yes vs. no) | 1.134 (0.755-1.705) | 0.544 | 0.989 (0.590-1.659) | 0.967 |
| gender (male vs. female) | 1.926 (1.376-2.698) | 0.001 | 1.853 (1.278-2.688) | 0.001 |
| age | 1.011 (0.995-1.027) | 0.167 |  |  |
| Cancer Stage (IV vs. III) | 1.544 (1.102-2.164) | 0.012 | 1.459 (1.023-2.081) | 0.037 |
| NSAIDs use (yes vs. no) | 2.731 (1.643-4.54) | 0 | 2.306 (1.355-3.925） | 0.002 |
| Chemotherapy (yes vs. no) | 1.01 (0.709-1.439) | 0.955 |  |  |
| Hypertension (yes vs. no) | 1.305 (0.946-1.801) | 0.105 |  |  |
| coronary heart disease (yes vs. no) | 1.04 (0.657-1.645) | 0.867 |  |  |
| Smoking history (yes vs. no) | 1.365 (0.963-1.936) | 0.08 | 1.061 (0.720-1.563) | 0.766 |
| EGFR-TKI regimen (ref: gefitinib) |  | 0.566 |  | 0.652 |
| Afatinib | 1.011 (0.569-1.797) | 0.97 | 0.725 (0.388-1.355) | 0.314 |
| Erlotinib | 1.158 (0.632-2.12) | 0.635 | 1.079 (0.579-2.008) | 0.811 |
| Icotinib | 0.701 (0.383-1.285) | 0.251 | 0.765 (0.414-1.415) | 0.393 |
| Osimertinib | 0.991 (0.611-1.61) | 0.972 | 0.893 (0.546-1.462) | 0.653 |
| WBC | 1.053 (0.996-1.112) | 0.067 | 1.026 (0.967-1.090) | 0.391 |
| HGB | 0.999 (0.991-1.006) | 0.759 |  |  |
| PLT | 1 (0.998-1.002) | 0.825 |  |  |
| ESR | 1.008 (0.998-1.019) | 0.115 |  |  |
| hsCRP | 1.005 (1-1.009) | 0.053 | 1.005 (1.000-1.011) | 0.063 |
